# Muscæ Volitantes

**Published:** 1892-06

**Authors:** A. D. Williams


### THE SIGNIFICANCE OF MUSCÆ VOLITANTES.

People are often frightened almost out of their wits by the sudden appearance of flying specks before their eyes; sometimes they are only one or two, but often thousands of them can be seen, particularly when a person looks toward a white surface, as white clouds, white houses, white pavements, or towards water surface. These flying specks are mostly small points, connected one with another by fine lines, and the points often presented a beaded appearance. At first, persons are likely to try to knock them away, thinking it is something before their eyes. They come usually in both eyes at the same time. They may diminish or increase in number at times, but rarely ever disappear entirely. They usually have a fixed position in the field, but occasionally they move or float about to a limited extent. They never interfere with vision by settling over objects looked at. They are invisible with the ophthalmoscope. Their nature is not well understood. The explanation usually given is that they are opaque points in the vitreous humor, which throw shadows upon the retina and thus become visible. Badly focused eyes are most likely to be troubled with muscæ volitantes. They signify nothing serious so long as they are mere points, connected by fine lines, and do not interfere with the acuteness of vision. Treatment is more than useless. If the eyes are out of focus, proper glasses should be selected. It is important that the patient should ignore their presence entirely; should avoid seeing them as much as possible and let them alone. Large floating masses before the eyes, which swim around and often obscure vision, are the result of serious disease and should be promptly looked after.—A. D. WILLIAMS, M. D.

